# Intrinsic disorder in the partitioning protein KorB persists after co-operative complex formation with operator DNA and KorA

**DOI:** 10.1042/BCJ20170281

**Published:** 2017-08-31

**Authors:** Eva I. Hyde, Philip Callow, Karthik V. Rajasekar, Peter Timmins, Trushar R. Patel, Giuliano Siligardi, Rohanah Hussain, Scott A. White, Christopher M. Thomas, David J. Scott

**Affiliations:** 1 School of Biosciences, University of Birmingham, Birmingham B15 2TT, U.K.; 2 Institut Laue Langevin, 71 avenue des Martyrs, CS 20156, 38042 Grenoble Cedex 9, France; 3 Diamond Light Source Ltd, Harwell Science and Innovation Campus, Didcot, Oxfordshire OX11 0DE, U.K.; 4 School of Biosciences, University of Nottingham, Sutton Bonington Campus, Leicestershire LE12 5RD, U.K.; 5 ISIS Neutron and Muon Spallation Source and Research Complex at Harwell, Rutherford Appleton Laboratory, Oxfordshire, U.K.

**Keywords:** circular dichroism, intrinsically disordered proteins, protein–DNA interactions, protein–protein interactions, small-angle scattering, transcription regulation

## Abstract

The ParB protein, KorB, from the RK2 plasmid is required for DNA partitioning and transcriptional repression. It acts co-operatively with other proteins, including the repressor KorA. Like many multifunctional proteins, KorB contains regions of intrinsically disordered structure, existing in a large ensemble of interconverting conformations. Using NMR spectroscopy, circular dichroism and small-angle neutron scattering, we studied KorB selectively within its binary complexes with KorA and DNA, and within the ternary KorA/KorB/DNA complex. The bound KorB protein remains disordered with a mobile C-terminal domain and no changes in the secondary structure, but increases in the radius of gyration on complex formation. Comparison of wild-type KorB with an N-terminal deletion mutant allows a model of the ensemble average distances between the domains when bound to DNA. We propose that the positive co-operativity between KorB, KorA and DNA results from conformational restriction of KorB on binding each partner, while maintaining disorder.

## Introduction

A large proportion of proteins have stretches of 50 residues or more that do not fold but exist as dynamic ensembles with little or no rigid secondary structure (reviewed in ref. [1]). These intrinsically disordered regions (IDRs) act as sites for interactions with multiple targets and are over-represented in classes of proteins that form network hubs, such as transcription factors and signalling proteins. Proteins with such regions, although extremely important for cellular processes, are difficult to characterise structurally, as they cannot be crystallised and are often too large for NMR studies and too small for CryoElectron Microscopy. One such protein is the ParB family protein, KorB, from the low copy-number IncP1 plasmid RK2 that is required for plasmid DNA partitioning [2] and for transcription repression [3].

To ensure the correct partitioning of chromosomes before cell division, most bacteria use a type Ia partitioning system consisting of a DNA-binding protein from the ParB family; a filament-forming ATPase from the ParA family, and a *cis*-acting, centromere-like site [4]. Such systems are also required for the active partitioning of many low copy-number plasmids, and plasmids have been widely used as tractable models for chromosomal partitioning in bacteria. Surprisingly, the structural basis of this critical process is largely unknown; while the ParA proteins are homologous and structures of a few intact ParA proteins have been determined [5–7], ParB proteins vary structurally and are more difficult to study as they contain multiple domains with highly flexible linkers [8]. KorB is one of the best characterised ParB proteins. It binds specifically to several sites on the plasmid DNA [9,10] and to the RK2 homologue of ParA, IncC, stimulating the latter's ATPase activity [11]. The partitioning complex, containing KorB, IncC and DNA, then moves to the appropriate positions in the bacterial cell, so that the plasmids are inherited by both daughter cells on cell division. KorB is also a weak transcriptional repressor; however, in the presence of other plasmid repressors, such as KorA or TrbA, co-operative repression is observed [12–15]. This co-operativity allows stringent gene regulation at low concentrations of the proteins, minimising the burden the plasmid places on its host. KorA and KorB acting together control expression of all the genes involved in plasmid replication and partition, including the central control operon encoding KorA and the partitioning proteins, KorB and IncC. Intriguingly, although the centre-to-centre distance between the KorB and KorA operators is always 33 base pairs [3], co-operativity between the two proteins can occur at many different distances between their binding sites [12].

**Figure 1.**
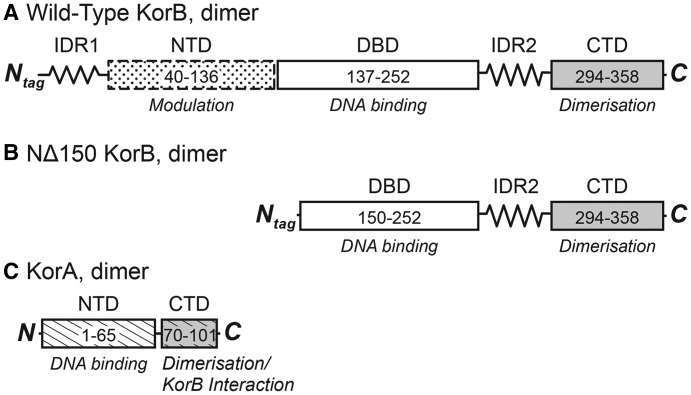
Domain organisation of KorB and KorA. (**A**) The domain organisation of KorB: there are two regions of intrinsic disorder in KorB, IDR1, the N-terminal tail, residues 1–54, and IDR2, a linker, residues 253–294 [21]. Structures of the DBD (residues 137–252) [17] and the C-terminal domain (CTD, residues 294–358) [16] have been determined previously by X-ray crystallography. The region between residues 40–252 is homologous to Spo0J from *T. thermophilus* [19] and *H. pylori* [20], the structures of which have been determined. (**B**) The domain organisation of (NΔ150)KorB; this mutant lacks the first 150 amino acid residues of KorB and so contains most of the DBD, the entire linker (IDR2) and CTD of KorB. (**C**) The domain organisation of KorA; there is a short linker between the N-terminal DBD (NTD residues 1–65) and the C-terminal dimerisation domain (CTD, 70–101). The latter domain is homologous to the C-terminal of TrbA [44].

KorB is a homodimer with each subunit consisting of 358 amino acid residues with four distinct segments (Figure 1A). Two regions of KorB have been crystallised, namely the central DNA-binding domain (DBD, residues 137–252) and the C-terminal dimerisation domain (residues 294–358). The C-terminal domain is a five-stranded antiparallel β-barrel [16], while the central region contains two α-helical subdomains, both of which interact with operator DNA [17]. This central region has a similar structure to the DNA-binding domains of other Class Ia ParB proteins [8,18], but the DNA contacts differ. The N-terminal region from residues ∼40–137 is homologous to Spo0J from *Thermus thermophilus* [19] and Spo0J from *Helicobacter pylori* [20], both of which have been crystallised with their DNA-binding regions. The region from *T. thermophilus* is largely folded in the crystal structure, whereas that from *H. pylori* has two short helices with varied loops that differ between different monomers in the same unit cell, so this region may be only partially folded in solution. Two further regions of KorB are predicted from the amino acid sequence to be intrinsically disordered: one between the DNA-binding domain and the C-terminal dimerisation domain (residues 253–293), and the other at the N-terminus (residues 1–54). These structural predictions are consistent with our previous studies of the free protein and deletion mutants, using NMR spectroscopy, circular dichroism (CD) spectroscopy and small-angle X-ray scattering (SAXS) [21]. Our previous study also showed that the domains of free KorB act independently, and that the protein exists in a wide range of dynamic conformations [21]. We have also examined the interaction of KorA with KorB and shown that this is mediated by the C-terminal dimerisation domain of KorA (Figure 1C) [14], as expected from its homology with TrbA [15].

To understand how KorB mediates both its DNA binding and its co-operative mode of action with KorA, we here use small-angle neutron scattering (SANS) experiments, together with CD and NMR spectroscopy, to selectively probe the conformation of KorB in binary and ternary complexes with KorA and DNA. To obtain further insights into these interactions, we have also used SANS to examine the corresponding complexes of a deletion mutant of KorB, (NΔ150)KorB (Figure 1B). This mutant lacks the N-terminal 150 amino acids of KorB and so exists as a homodimer of the two domains that have been crystallised and the unstructured linker between them, allowing fuller modelling of the mutant.

SANS is particularly useful for studying macromolecular complexes as different nuclei, particularly deuterons and protons scatter neutrons with different phase and intensity; in contrast with SAXS, where the scattering is simply proportional to the number of electrons [22]. DNA and proteins scatter neutrons with different average intensity and, at low resolution, where the scattering of the components can be considered as homogeneous, it is possible to match the scattering of selected components within a complex using appropriate levels of solvent deuteration (contrast matching) [23,24]. A solvent containing 40% D_2_O matches the average scattering length density of proteins at natural proton abundance, and thus proteins are not detected in a SANS experiment in this solvent, while nucleic acids typically match out at 65–70% D_2_O. Increasing the deuteration level of a protein by expressing it in specific isotopically labelled media moves the match point of that protein to higher D_2_O concentrations. Solvent matching has been used previously to examine macromolecules selectively in binary complexes; we have extended this technique to examine KorB directly in a ternary complex with KorA and DNA, by partial deuteration of KorA to give the same match point as the DNA.

## Experimental procedures

### Protein expression and purification

KorB, (NΔ150)KorB and the C-terminal domain of KorB were expressed as His-tagged fusions in *Escherichia coli* BL21(λDE3) cells and purified as described previously [21]. KorA was expressed in *E. coli* BL21(λDE3) cells, without a tag, and purified as before [14]. KorB and KorA were partially deuterated by the expression in *E. coli* grown in M9 minimal medium with ∼70% (v/v) ^2^D_2_O/30% (v/v) H_2_O solvent; for NMR studies, the growth medium contained ^15^N-NH_4_Cl and ^13^C_6_-glucose as the sole nitrogen and carbon sources, respectively. To obtain KorA with the same match point as DNA, it was expressed in *E. coli* grown in M9 minimal medium with 46% (v/v) ^2^D_2_O/54% (v/v) H_2_O solvent [25]. Protein concentrations were based on their absorbance at 280 nm, using predicted molar absorbance coefficients [26], and are given as dimers throughout.

### DNA oligonucleotides

DNA oligonucleotides were purchased from MWG or Sigma and purified by HPLC. DNA concentrations were based on the manufacturers' analysis of yield. As the footprint of KorB on its operator is over 22 base pairs [9], to study the binary complex of a dimer of KorB with its operator, we used a 26 base pair DNA fragment containing the 13 bp consensus O_B_ site and the most favourable flanking bases:

O_B_: 5′-CGAGAAT**TTTAGCGGCTAAA**AAGGGC-3′

To study the ternary complex of a dimer of KorB with a dimer of KorA and double-stranded DNA, we used a 57 base pair DNA sequence based on the central control operon, containing operators for both KorA and KorB, O_A_O_B_:

5′-GGACGAGT**TTTAGCGGCTAAA**GGTGTTGACGTGCGAGAAAT***GTTTAGCTAAAC***TTCG-3′

The KorB consensus binding sequence is in bold and underlined, and the KorA consensus binding sequence is in bold and italics.

### NMR spectroscopy

2D ^15^N-^1^H TROSY NMR spectra [27] of ^15^N/^2^D-labelled KorB and its complexes (∼0.1 mM dimer) were collected on a Varian 800 MHz spectrometer using a triple resonance cryoprobe, in 10 mM sodium phosphate buffer (pH 7.0), 100 mM NaCl and 0.1 mM EDTA at 30°C. For the C-terminal domain of KorB, an HSQC spectrum was collected on a Bruker 500 MHz spectrometer, under similar conditions. Spectra were transformed using NMRPipe [28] and examined and plotted using the CCPN [29] software.

### CD spectroscopy

CD spectra were measured with an Applied Photophysics Chirascan Plus instrument on B23 at the Diamond Light Source (Oxfordshire, U.K.) or a JASCO 1500 instrument, with 3–10 µM KorA, KorB and O_A_O_B_ DNA in 10 mM sodium phosphate buffer (pH 7.0), 100 mM NaClO_4_ and 0.1 mM EDTA at 25°C in 0.01 cm Hellma-fixed cuvettes. Spectra were taken at 185–325 nm with a scan speed of 37 nm/min, 2–4 scans, and a bandwidth of 1 nm. The secondary structures of the proteins were analysed from the CD data using the CONTINLL algorithm [30] within the CDApps suite of programmes [31] with the SP37 database set of 37 soluble proteins.

### Small-angle X-ray scattering

SAXS data were collected either at beamline X33 at DESY, Hamburg, or on beamline ID14-3 ESRF, Grenoble. Proteins and complexes (3–15 mg/ml, ∼0.1–0.5 mM) were dialysed into 10 mM Tris–HCl (pH 7.0), 100 mM NaCl and 0.1 mM EDTA (Buffer A). Data were collected at an X-ray wavelength of 1.54 Å, at 20°C. Each protein or complex was measured at three different concentrations to monitor for aggregation. Samples were measured in flow mode to minimise any radiation damage, with 10 measurements taken over 2 min. The data were confirmed by SEC-SAXS, at Beamline BM29 at ESRF, Grenoble. Fifty microlitres of ∼0.1 mM of the proteins or complexes (3–10 mg/ml) were loaded onto a Superdex200 increase (3.2/300) column equilibrated with the above buffer, which was coupled to the SAXS flow cell, and measurements were taken as above. The scattering patterns were typically recorded in the range of momentum transfer 0.007 < *q* < 0.5 Å^−1^ (*q* *=* 4*π* sin(*θ*)/*λ*, where 2*θ* is the scattering angle and *λ* is the X-ray wavelength). The scattering of the dialysis buffer was subtracted from that of the macromolecular solution. The data from the different concentrations were processed and merged using standard procedures in PRIMUS [32].

### Small-angle neutron scattering

SANS data were collected on beamline D22 at the Institut Laue Langevin, Grenoble, France. Additional confirmatory measurements were made on LOQ at ISIS Spallation Neutron and Muon Source, Oxfordshire, U.K. Proteins and complexes (∼0.1 mM dimer final concentration, ∼10 mg/ml) were dialysed into Buffer A, made either in 100% (v/v) H_2_O or in 100% (v/v) D_2_O. The samples in H_2_O and D_2_O were mixed in the appropriate ratio to obtain 0, 40, 65 and 100% (v/v) D_2_O solvent and left to equilibrate. Sample transmissions were collected at 20°C at all contrasts for 2 min per sample, while scattering data were collected for 30 min, 4 h each, at detector distances of 2 and 5.6 m. An 8 Å wavelength was used, giving the *q* range of 0.012–0.15 Å^−1^. Data reduction used standard routines with the GRASansP software (Dewhurst, 2006: www.ill.fr/lss/grasp/grasp_main.html).

### Data analysis of small-angle scattering data

For both SAXS and SANS, the forward scattering *I*(0) and the radii of gyration *R*_g_ were evaluated using the programme PRIMUS [32] from the Guinier approximation at low angles [33] and from the entire scattering pattern using the indirect Fourier transform package GNOM [34], which also provides the maximum dimension of the particle *D*_max_ and the pairwise distance distribution function *P*(*r*). The scattering data of the complexes were also analysed in the form of a normalised Kratky plot of *I*(*q*)/*I*(0)](*qR*_g_)^2^ vs*. qR*_g_ [35]. The match points of the partially deuterated KorB and KorA proteins were determined by plotting the square root of the forward scattering intensity (√*I*(0)) vs. (v/v) % D_2_O in the solvent. The scattering density and volumes of KorB, (NΔ150)KorB and O_B_ for SAXS and SANS in H_2_O and D_2_O were calculated using the programme MULCh [36]. The determination of molecular mass was performed first by the Porod analysis [37]. This provides an absolute measure of molecular volume from which a molecular mass can be determined providing there is knowledge of the particles’ partial specific volume, which for proteins can be calculated from the amino acid sequence. Porod analysis does not work for extended molecules, and as such it was applied only to the *apo* proteins where the conformation is more compact. The remaining molecular mass was determined by using comparison of the *I*(0) values with those of the *apo* proteins, taking account of relative concentration and neutron contrast with the solvent.

## Results

### The C-terminal domain of KorB remains mobile in its ternary complex with KorA and O_A_O_B_ DNA

**Figure 2.**
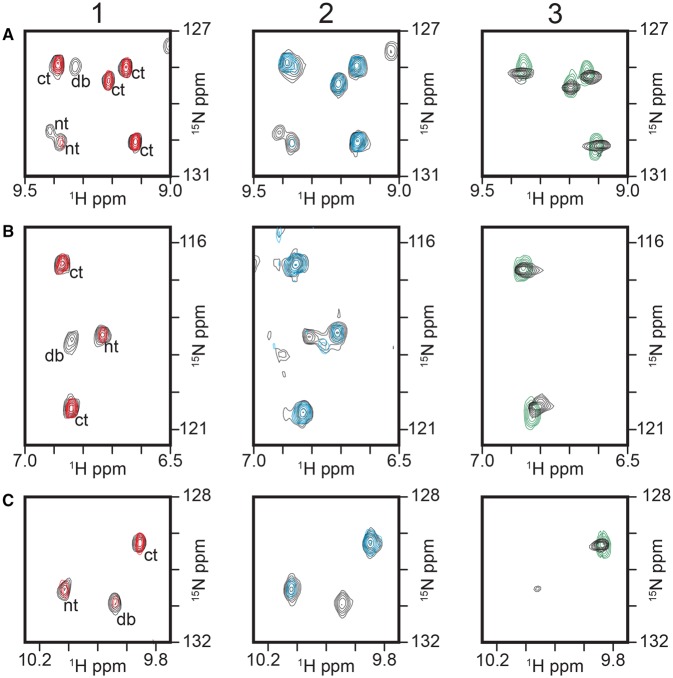
Sections of the ^15^N-^1^H TROSY spectra of ^15^N/^2^D KorB complexes, taken at 800 MHz, overlaid with those of the free protein. (Top and middle) Box A and Box B: two regions of the spectrum with peaks from different domains of the protein; (bottom) Box C: the three tryptophan indole NH peaks. The peaks are labelled in panel 1, as coming from the N-terminal domain (nt), the DNA-binding domain (db) and the C-terminal domain (ct). (Left) Panel 1: ^15^N/^2^D KorB (∼0.12 mM dimer) in the presence of 0.75 molar equivalent O_B_ DNA (red) overlaid on the spectrum of free KorB (black). (Centre) Panel 2: ^15^N/^2^D KorB in the presence of 2 molar equivalents unlabelled KorA (blue) overlaid on the spectrum of free KorB (black). (Right) Panel 3: ^15^N/^2^D KorB in the presence of 1 equivalent unlabelled KorA and 1 equivalent of O_A_O_B_ DNA (green) overlaid with the spectrum of ^15^N-labelled C-terminal domain (residues 298–358) of KorB (black) taken at 500 MHz.

We initially used ^15^N-^1^H TROSY NMR experiments to examine KorB in its complexes. The KorB protein was ^15^N-labelled and its spectrum monitored on titration with O_B_ DNA, a 26 bp fragment containing the KorB-binding site, or with unlabelled KorA (selected peaks shown in Figure 2 and full spectrum in Supplementary Figure S1). As only the KorB is ^15^N-labelled, the signals observed come only from KorB.

We have previously shown that ^15^N/D-labelled KorB gives a relatively sharp NMR ^15^N-^1^H TROSY spectrum, despite its 80 kDa molecular mass, and that the spectra of the separate folded domains can be overlaid on that of the full protein [21]. This shows that in the free protein, the domains are independent and allows assignment of the resolved signals to different regions of the protein. The three tryptophan indole NH resonances in KorB are readily identified, as they resonate near 130 ppm (^15^N) and 10 ppm (^1^H) and each comes from a separate domain (Figure 2, row C). The remaining resolved peaks in the spectrum have been assigned to either the N-terminal (nt) or the DNA-binding region (db) or the C-terminal region of the protein (ct), but the exact residues have yet to be assigned; two representative resolved regions are shown in Figure 2 in rows A and B.

On addition of increasing amounts of O_B_ DNA to KorB, selected peaks in the spectrum decreased in intensity and then were no longer observed (Figure 2, left panel and Supplementary Figure S1, panel 1). The signals for NH groups in the DNA-binding domain (db) of free KorB are, in general, of lower intensity than those from the other domains and disappeared at lower concentrations of DNA than those from the N-terminal region (nt), showing that this region is most affected by DNA binding; while peaks from NH groups in the C-terminal region (ct) were unaffected by the addition of DNA.

On addition of 1 equivalent of KorA to ^15^N-labelled KorB, there was little effect on the NMR spectrum of the latter, but on addition of 1 equivalent on the second equivalent, again the resonances from the DNA-binding domain and the N-terminal domain disappeared (Figure 2, central panel and Supplementary Figure S1, panel 2). This ratio is consistent with our previous NMR titration of ^15^N-labelled KorA with KorB that showed full binding at a ratio of KorB : KorA of 1 : 2 [14]. The ^15^N-^1^H TROSY spectra of KorB in the two complexes KorB-O_B_ 1 : 1 and KorB : KorA at a ratio of 1 : 2 show the same subset of peaks remaining in the spectrum (Supplementary Figure S1).

In the ternary complex of KorB with KorA and O_A_O_B_ DNA, a 57 bp DNA fragment that contains operators for both KorA and KorB, the spectrum contains resolved signals solely at positions nearly identical with those of the isolated C-terminal dimerisation domain (Figure 2, right panel and Supplementary Figure S1, panel 3), with no resolved signals from the DNA-binding or N-terminal domain. A few additional sharp signals are seen in the centre of the ^1^H spectrum of the ternary complex, many of which are also seen in the ^15^N-spectrum of (NΔ150)KorB and so are probably from the linker between the central and C-terminal domains. This spectrum shows conclusively that the C-terminal domain of KorB remains independently mobile within the 120 kDa complex containing both DNA and KorA, and is unaffected by DNA and KorA binding; however, little can be deduced about the remainder of the protein from the NMR spectra.

### The secondary structure of KorB is unchanged on complex formation with DNA and KorA

**Figure 3.**
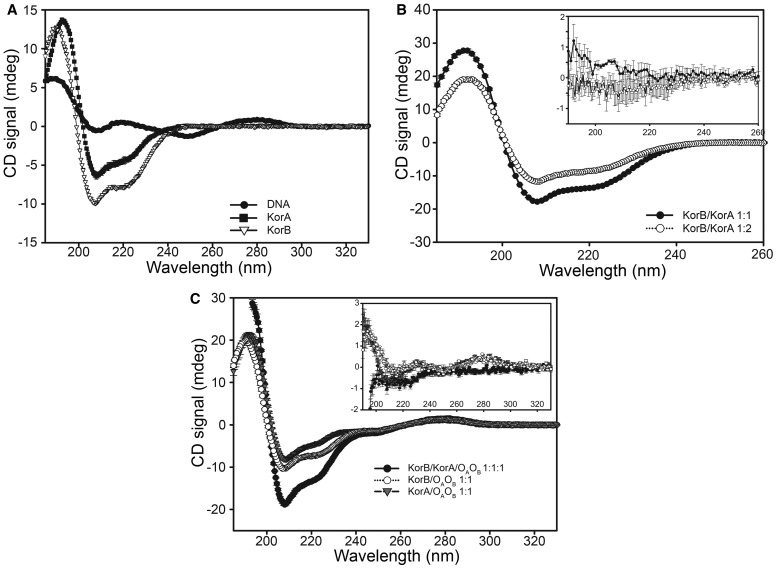
CD spectra of KorB, KorA, O_A_O_B_ DNA and their complexes. (**A**) CD spectra of the individual components; KorB (10 µM) (white triangles), KorA (10 µM) (black squares) and O_A_O_B_ DNA (10 µM) (black circles). Spectra were taken in a 0.01 cm pathlength cuvette. Error bars show the differences between two technical repeats. (**B**) CD spectrum of KorB–KorA complexes; KorB/KorA at 1 : 1 (10 µM each) (black circles); and KorB/KorA 1 : 2 [KorB (10 µM)  + KorA (5 µM)] (white circles). Spectra were taken in a 0.01 cm pathlength cuvette. Error bars show the differences between two technical repeats. Inset—the difference between the measured spectra and those calculated from the sum of the spectra of the separate proteins, with their associated errors. (**C**) CD spectra of DNA complexes; KorA/O_A_O_B_ DNA (10 µM each) (grey triangles), KorB/O_A_O_B_ DNA (10 µM each) (white circles); KorB /KorA/O_A_O_B_ DNA (10 µM each) (black circle). Spectra were taken in a 0.01 cm pathlength cuvette. Error bars show the differences between two technical repeats. Inset—for the binary complexes, the difference between the measured spectra and that calculated from the sum of the spectra of the separate components, with their associated errors. For the ternary complex, the expected spectrum was calculated from the sum of the spectra of the two binary complexes (KorB/O_A_O_B_ + KorA/O_A_O_B_) minus that of the free O_A_O_B_ DNA.

To determine the effect of complex formation on the secondary structure of KorB, we examined the CD spectra of the complexes of KorB with DNA and KorA. Figure 3A shows the CD spectra of free KorB, KorA and O_A_O_B_ DNA, at 10 µM concentration. In the near-UV region of the spectrum, near 280 nm, only signals from the DNA can be seen under these conditions, and the signals from protein are ∼10–100-fold weaker; conversely in the far-UV region, at ∼220 nm, the signals from the protein are much larger than those from the DNA. The secondary structure composition of free KorB, estimated from the CD spectrum using the CONTILL algorithm (Supplementary Table S1), agrees well with our previous study [21], while that of KorA suggests slightly higher α-helical content than the crystal structure of the free protein [38].

Figure 3B shows the CD spectra of KorA at 10 µM in the presence of KorB at 5 µM, i.e. at 2 : 1 KorA : KorB, and with KorB at 10 µM, i.e. at 1 : 1 KorA : KorB. In each case, the CD spectrum of the KorA : KorB mixture was the same as that simulated by adding the spectrum of KorA to that of KorB, (inset) indicating that there are no secondary structure changes upon complex formation between the two proteins.

Figure 3C shows the spectra of the O_A_O_B_ DNA in the presence of 1 equivalent of KorB or 1 equivalent of KorA or both KorA and KorB. For KorA and KorB complexes, the inset shows the differences between the observed spectrum and that calculated by adding the spectra of free DNA to that of free protein. For the KorB/O_A_O_B_ complex, small but highly reproducible differences are seen between the CD spectrum of the complex and the sum of the free components at 280 nm, but at 210–230 nm the difference spectrum is zero (Figure 3C, inset), showing that the secondary structure of KorB is unaffected by the addition of DNA. Since the spectrum at 280 nm is solely due to the DNA, the changes here are ascribed to changes in the tilt angle of the bases of the DNA on KorB binding, showing that there is some bending of the DNA on protein binding.

On adding KorA to O_A_O_B_ DNA, the same changes are seen in the near-UV CD spectrum at 280 nm as on KorB binding (Figure 3C, inset), suggesting that both proteins alter the DNA structure in the same way. There is a slight increase in the CD signal of the KorA/O_A_O_B_ complex at 210 nm, suggesting that there may be a small increase in α-helical content of KorA; however, the noise in this region of the spectrum is greater than that in the near-UV regions, and this change is small and not reproducible. No change in the secondary structure was observed in the crystal structure of KorA on binding O_A_, but the two DNA-binding domains moved relative to the dimerisation domain [38]. On addition of KorA and KorB and O_A_O_B_ at 1 equivalent each, the spectrum observed is very similar to that expected from the sum of the effects of each protein on the DNA separately (i.e. spectra KorB : DNA plus KorA : DNA minus free DNA; Figure 3C, inset). There is no increase in signal at 280 nm over that from each protein separately, showing that the effect of each protein on the bending of the DNA is additive in the ternary complex. At 210 nm, there is a very slight increase in intensity over that calculated. This effect is small and not reproducible (∼5% total signal), suggesting that any change in secondary structure of the proteins on ternary complex formation is small.

### KorB has a greater proportion of extended conformers in binary and ternary complexes of DNA and KorA; however, it retains some random coil structure

To examine the effect of complex formation on the overall conformation of KorB, we turned to small-angle scattering. Initial experiments confirmed that the neutron scattering of free ^1^H-KorB in both H_2_O and D_2_O, and of ^2^D-KorB gave similar pairwise distance distribution functions (*P*(*r*) vs. *r*) both to each other and to the distribution from the X-ray scattering data (Supplementary Figure S2). The pairwise distribution function shows the proportion of two scattering particles at a distance (*r*) apart within the molecule. For the free protein, only a small difference is expected between the SAXS and SANS measurements, due to the observation (or matching) of the hydration layer around the protein [39]. Calculated molecular masses were consistent with the monomer molecular masses within experimental error (Table 1). Thus, these results, importantly, confirm that there is no effect on the conformation of the protein on changing the deuteration level of the solvent or on protein deuteration.

**Table 1:** Analysis of scattering data of WT KorB or (NΔ150)KorB, and for each of the complexes, at different contrasts Left — Guinier analysis of the very low angle scattering, from the programme Primus [32], *qR*_g_ range used for the Guinier analysis, radius of gyration, *R*_g_, and initial intensity *I*(0). Right — the same parameters from the indirect Fourier transform programme GNOM [45] for the whole data, with the *q* range and the *D*_max_, maximum dimension, used for the calculations.

Sample	Method	% D_2_O	Guinier			Concentration	Mwt_exp_	Mwt_calc_	*q* range (Å^−1^)	Real space		
			*qR*_g_ range	*R*_g_ (Å)	*I*(0)					*R*_g_ (Å)	*I*(0)	*D*_max_ (Å)
^1^H-KorB	SAXS	0	0.42–1.28	57.6 ± 0.02	771 ± 2	7/14^1^	81.4 ± 5.0^2^	83.0	0.007–0.48	58.4 ± 0.06	761 ± 0.88	200
	SANS	0	*1.02–1.57*^3^	*49.7* ± *1.6*	*0.40* ± *0.02*	10.45	76.7 ± 10.0^4^	83.0	0.020–0.236	59.8 ± 0.4	0.41 ± 0.01	200
	SANS	100	*1.02–1.63*^3^	*49.3* ± *0.2*	*1.211* ± *0.007*	10.86	116 ± 30.0^4^	83.0	0.020–0.236	58.7 ± 0.5	1.38 ± 0.01	195
^2^D-KorB	SANS	100	0.84–1.25	52.5 ± 1.2	0.67 ± 0.01	4.00	78.9 ± 15.0^4^	83.0	0.016–0.3	54.2 ± 0.5	0.67 ± 0.05	180
^1^H-KorB-O_B_	SAXS	0	0.80–1.24	49.8 ± 0.02	22.2 ± 0.03				0.012–0.25	51.8 ± 0.2	225.0 ± 0.9	200
	SANS	0	0.78–1.27	49.7 ± 1.8	0.519 ± 0.003	9.10	117 ± 20.0^4^	98.9	0.013–0.204	54.6 ± 0.3	0.520 ± 0.002	205
	SANS	100	0.69–1.32	56.5 ± 0.3	0.300 ± 0.001	5.20	90 ± 12.0^4^	98.9	0.013–0.204	57.2 ± 0.2	0.300 ± 0.001	200
	SANS	65	0.96–1.32	61 ± 4	0.048 ± 0.002	6.50	71.0 ± 12.0^4^	83.0^5^	0.013–0.194	62.6 ± 1.7	0.045 ± 0.001	195
^1^H-KorB/^2^D-KorA 1 : 1	SANS	100	0.98–1.36	66.6 ± 0.3	0.720 ± 0.004	3.32^6^	84.0 ± 9.0^4^	83.0^5^	0.0145–0.189	69.5 ± 0.2	0.700 ± 0.003	230
^1^H-KorB/^2^D-KorA 1 : 2	SANS	100	0.54–1.27	63.8 ± 0.7	0.380 ± 0.003	7.06^6^	94.4 ± 8.0^4^	83.0^5^	0.012–0.255	70.6 ± 0.5	0.360 ± 0.002	260
^2^D-KorB/^1^H-KorA 1 : 1	SANS	40	0.77–1.30	67.6 ± 0.8	0.810 ± 0.007	7.12^6^	82.0 ± 10.0^4^	83.0^5^	0.011–0.192	64.7 ± 0.4	0.77 ± 0.04	235
^2^D-KorB/^1^H-KorA 1 : 2	SANS	40	0.839–1.32	64.4 ± 0.9	0.650 ± 0.007	7.12^6^	73.6 ± 10.0^4^	83.0^5^	0.013–0.25	66.4 ± 0.5	0.638 ± 0.04	230
^1^H-KorB/46% ^2^D-KorA/O_A_O_B_	SANS	65	0.645–1.63	66 ± 2	0.101 ± 0.006	7.79^6^	120 ± 30.0^4^	98.0^5^	0.009–0.264	64 ± 1	0.082 ± 0.03	195
^1^H-(NΔ150)KorB	SAXS	0	0.734–1.26	41.5 ± 0.01	255.1 ± 0.7				0.018–0.50	41.5 ± 0.004	249.0 ± 0.3	135
	SANS	0	0.794–1.38	38.5 ± 0.8	0.271 ± 0.005	6.20	48.4 ± 8.0^4^	51.6	0.02–0.26	40.5 ± 0.6	0.27 ± 0.04	130
	SANS	100	0.686–1.27	36.8 ± 0.4	0.144 ± 0.001	6.20	48.5 ± 9.0^4^	51.6	0.0177–0.35	35.4 ± 0.4	0.140 ± 0.009	139
^1^H-(NΔ150)KorB/O_B_	SAXS	0	0.651–1.04	39.9 ± 0.02	350.7 ± 0.9				0.016–0.22	40.1 ± 0.007	348.8 ± 0.4	145
	SANS	0	0.48–1.29	36.2 ± 0.6	0.205 ± 0.002	6.62	66.7 ± 9.0^4^	67.5	0.0132–0.30	37.0 ± 0.5	0.200 ± 0.002	125
	SANS	65	0.75–1.36	43 ± 3	0.035 ± 0.002	6.62^6^	51.9 ± 8.0^7^	51.6^5^	0.0133–0.334	42 ± 1	0.033 ± 0.007	135
	SANS	100	0.674–1.3	38.0 ± 0.35	0.161 ± 0.001	6.62	76 ± 10.0^7^	67.5	0.017–0.308	38.3 ± 0.3	0.158 ± 0.008	135
^1^H-(NΔ150)KorB/46% ^2^D-KorB/O_A_O_B_	SANS	65	0.52–1.23	48 ± 4	0.033 ± 0.003	6.62^6^	51.7 ± 8.0^7^	51.6^5^	0.008–0.214	43 ± 2	0.029 ± 0.001	140

1 Data taken from two merged datasets at different concentrations. *I*(0) in the Guinier region was calculated from the 7 mg/ml dataset, whereas the *I*(0) and *R*_g_ from the GNOM analysis were computed from the merged datasets.

2 Molecular mass calculated from comparison with a 5 mg/ml BSA standard.

3 For free KorB, the SANS measurements were not collected at sufficiently low angles to obtain a Guinier plot below *qR*_g_ = 1.3; hence, the values obtained for *R*_g_ are much lower than from the transformed data in GNOM.

4 Molecular masses derived from Porod analysis.

5 Calculated visible molecular mass at given (v/v) D_2_O concentration.

6 Concentration of the visible component as this D_2_O concentration.

7 Molecular masses obtained by comparison of forward scatter of samples with those which were analysed by the Porod analysis.

**Figure 4.**
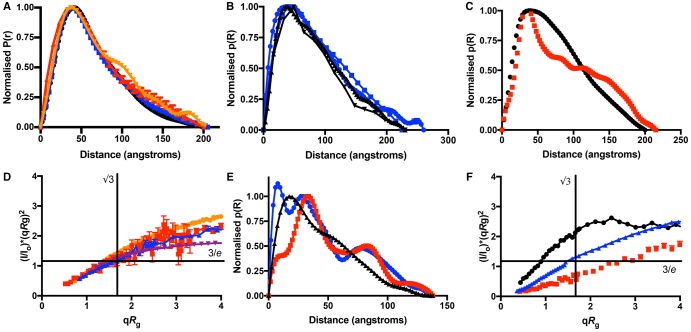
Pairwise distribution functions and normalised Kratky plots of KorB and (NΔ150)KorB within their binary and ternary complexes. (**A**) Pairwise distance distribution functions derived from SAXS studies of KorB/O_B_ in H_2_O (black) and from SANS studies of ^1^H-KorB/O_B_ in 0% ^2^D_2_O (H_2_O) (blue), 65% D_2_O (red) and in 100% ^2^D_2_O (orange). The functions have been normalised to give a maximum of one unit and error bars calculated by GNOM are shown. (**B**) Pairwise distance distribution functions derived from SANS for KorB within KorB/KorA complexes. ^1^H-KorB/^2^D-KorA/1 : 1 (black inverted triangles) and ^1^H-KorB/^2^D-KorA 2 : 1 (black triangles) both at 100% D_2_O, when the ^2^D-KorA is matched out; ^2^D-KorB/^1^H-KorA 1 : 1 (blue squares), ^2^D-KorB/^1^H-KorA 1 : 2 (blue circles), both at 40% D_2_O, when the ^1^H-KorA is matched out. The functions have been normalised to give a maximum of one unit and error bars calculated by GNOM are shown. (**C**) Pairwise distance distribution functions derived from SANS for ^1^H KorB in 0% D_2_O (H_2_O) (black), and ^1^H-KorB/46% ^2^D-KorA/O_A_O_B_ at 65% D_2_O (red) when both the KorA and the DNA are matched out. The functions have been normalised to give a maximum of one unit and error bars calculated by GNOM are shown. (**D**) Normalised Kratky plot of *I*(*q*)/*I*(0) * (*qR*_g_)^2^ vs*. qR*_g_ for free ^1^H-KorB in 100% D_2_O (purple), ^1^H-KorB/O_B_ at 65% D_2_O (blue), ^2^D-KorA/^1^H-KorB at 100% D_2_O (orange) and ^1^H-KorB/46% ^2^D-KorA/O_A_O_B_ at 65% D_2_O (red); only KorB will be visible under each of these conditions. The error bars calculated from those for the scattering intensity are shown. (**E**) Pairwise distance distribution function derived from SANS for free ^1^H-(NΔ150)KorB in 0% D_2_O (H_2_O) (black) and within ^1^H-(NΔ150)KorB/O_B_ at 65% D_2_O (blue), where the DNA is matched out and ^1^H-(NΔ150)KorB/46% ^2^D-KorA/O_A_O_B_ at 65% D_2_O (red) where both the KorA and the DNA are matched out. The function has been normalised to constant area, and error bars calculated by GNOM are shown. (**F**) Normalised Kratky plot of *I*(*q*)/*I*(0) * (*qR*_g_)^2^ vs*. qR*_g_ for (NΔ150)KorB in the same complexes as in **E**.

On adding O_B_ DNA, SAXS measurements show that the overall radius of gyration of the KorB/O_B_ complex, due to the scattering particles, is less than that of the free protein (Table 1). In SANS in either 100% (v/v) H_2_O or 100% (v/v) D_2_O, as in SAXS experiments, the KorB/O_B_ complex shows contributions from both the DNA and the protein, and the *P*(*r*) function is similar to that of the SAXS experiments (Figure 4A). Calculated molecular masses of the particles were all consistent with the DNA/protein complex (Table 1). However, importantly, at 65% D_2_O, DNA has similar scattering intensity to the solvent and so one observes only the protein component of the complex. The radius of gyration of the protein within the complex and its *P*(*r*) function can, therefore, be estimated from the scattering of KorB/O_B_ at 65% D_2_O. For the DNA-bound protein, the radius of gyration *R*_g_ is slightly larger (Table 1), and the *P*(*r*) function shows an overall shift to longer distances when compared with the free protein (Figure 4A,C), showing that the KorB protein within the DNA complex contains a greater proportion of more extended conformations than in the free state. To check this change is not due to D_2_O-dependent aggregation, we plotted the square root of the forward scattering intensity (√*I*_0_) vs. (v/v) % ^2^D_2_O in the solvent (Supplementary Figure S3A). This plot is linear as expected for a non-aggregating system, as are the Guinier plots for each contrast (Supplementary Figure S3B) [40]. This is also confirmed by the determination of the molecular masses, which are all consistent with the calculated values (Table 1). Hence, we are observing a genuine change in the range of KorB conformations when bound to DNA.

To examine KorB in the binary complex with KorA using SANS, KorA was 70% deuterated, so that its scattering matched ∼100% D_2_O solvent, and we measured scattering from complexes containing ^2^D-KorA : KorB in a 1 : 1 ratio, and also in a 2 : 1 ratio. By observing the scatter at 100% D_2_O, only KorB is observed, regardless of excess KorA. Both 1 : 1 and 2 : 1 complexes gave similar pairwise distribution curves (Figure 4B) with similar values for *R*_g_ and *D*_max_. The radius of gyration of KorB within the binary KorA complexes is again larger than that of free KorB and similar to that in the KorB/O_B_ complex (Table 1), and the pairwise distribution function of KorB in the KorA complexes has a similar envelope to that of the binary complex with O_B_ DNA; however, it is rather featureless, suggesting a broader distribution of conformations.

To check that the effects were not due to the high concentrations of D_2_O required for solvent matching the deuterated KorA, we also made 70% deuterated KorB and measured the scattering of 1 : 1 and 1 : 2 complexes of ^2^D-KorB/^1^H-KorA at 40% D_2_O, where the scattering from the protonated KorA is matched to the solvent. Since KorA is ∼25% of the size of KorB, at low resolution the fluctuations in contrast due to KorA are not observed, so again only the scattering from KorB is observed. At 40% D_2_O, the ^2^D-KorB/^1^H-KorA samples gave similar *R*_g_ measurements (Table 1) and pairwise distance distribution *P*(*r*) curves (Figure 4B) for the KorB within the complex to those where the KorA was deuterated and a complex of ^1^H-KorB/^2^D-KorA was observed at 100% D_2_O. The values of molecular mass determined from the data are all consistent with the expected calculated values (Table 1). Hence, the changes observed are due to the changes in conformation of KorB on KorA binding, rather than aggregation due to the deuteration of the solvent or the KorB. From these data, we can, therefore, deduce that the range of conformers that KorB adopts in its binary complexes with KorA again contains a greater proportion of more extended conformations to those of the free KorB, similar in manner to those in its complex with DNA.

Finally, to observe solely the KorB in ternary complex with KorA and double operator DNA, O_A_O_B_, we expressed KorA from a partially deuterated minimal medium, so that it had almost the same neutron match point as the DNA (Supplementary Figure S4A), at 65% D_2_O. Thus, at 65% D_2_O, only the protonated KorB protein is observed in the complex. The pairwise distance distribution function of KorB within the ternary complex is again shifted to longer conformations than that of the free protein, but is now less broad than in the binary complexes, suggesting that the protein conformation is better defined than in the binary complexes or the free protein (Figure 4C). There is one major peak at ∼36 Å, and then a series of smaller peaks at longer distances, rather than the wide distance distribution between 36 and 200 Å seen in the free protein.

To further examine the flexibility of KorB in its complexes, we calculated normalised Kratky plots of (Figure 4D) for free ^1^H-KorB in D_2_O, ^1^H-KorB/O_B_ at 65% D_2_O, ^2^D-KorA/^1^H-KorB at 100% D_2_O and the ternary complex at 65% D_2_O; only KorB will be visible under each of these conditions. By scaling *q* by *R*_g_ and *I*(*q*) by *I*(0), this plot is made dimensionless and independent of the particle mass and concentration [35]. For a folded protein, a single bell-shaped curve is expected, with a maximum at co-ordinates (√3, 3/*e*), whereas for a completely unfolded random coil, the values should continue increasing at high values of *qR*_g_. For the free KorB and all its complexes, the peaks for the reduced Kratky plots have maxima greater than 1.1 and these lie to the right of the *qR*_g_ = √3. This shows that KorB retains some unfolded character in all of these complexes.

### (NΔ150)KorB shows similar effects to KorB on binding DNA and in the ternary complex with KorA and DNA

To determine whether the changes in conformation observed on complex formation occur in the C-terminal or N-terminal half of KorB, we performed similar SANS experiments with complexes of an N-terminally deleted KorB, (NΔ150)KorB. This lacks the first 150 amino acids of KorB and binds O_B_ DNA more tightly than wild-type (WT) KorB [41]. While deuterated protein behaves functionally similarly to non-deuterated protein, (NΔ150)KorB appears to aggregate at high D_2_O concentrations, so the experiments could only be done at lower protein concentrations and we were not able to match out fully deuterated KorA in the binary complexes. Figure 4E shows the pairwise distribution functions for (NΔ150)KorB of (i) the free protein, (ii) (NΔ150)KorB in the binary complex with O_B_ DNA and (iii) (NΔ150)KorB in the ternary complex with 46% ^2^DKorA and O_A_O_B_ DNA; Table 1 lists the derived *R*_g_ and *D*_max_ values. As for WT KorB, (NΔ150)KorB the distance distribution function for the free protein is broad, but in the binary DNA complex the distance distribution is more well defined, giving a peak at 20 Å with a shoulder at 35 Å and a distinct second peak at 80 Å of about half of the intensity. In the ternary complex, the first peak is at 35 Å, as in the KorB ternary complex, with the second major peak at 78 Å similar to the (NΔ150)KorB/O_B_ binary complex. Figure 4F shows the normalised Kratky plots for (NΔ150)KorB and its DNA complexes, showing that, like full-length KorB, they all retain some unfolded character.

## Discussion

In the present study, we have investigated the highly flexible protein KorB in its complexes with KorA and DNA. Owing to the intrinsically disordered regions, KorB cannot exist as a single conformer, but only as a set of interconverting range of conformers. The CD spectra show that the secondary structure of KorB is minimally affected on complex formation. The protein remains disordered and mobile in its binary and ternary complexes, as shown by the Kratky plots of the complexes and, in particular, by the C-terminal domain remaining visible in the NMR spectrum of the ternary complex, with chemical shifts similar to those of the free domain.

### Model of the binary KorB–O_B_ complex

Using SANS and contrast matching, we show that the binding of DNA and/or KorA causes a change in the pairwise distribution function of KorB, to favour longer, more extended, conformers. In a SAXS study of a comparable ParB protein from *Mycobacterium tuberculosis*, a reduction in overall radius of gyration of electron scattering was observed on DNA binding [42] and, hence, it was proposed that the protein became more compact upon complex formation. A similar reduction in the overall radius of gyration was seen on KorB binding to DNA in the present study, using SAXS (Table 1). Using SANS experiments, however, we have shown that the protein itself adopts a greater proportion of more extended conformers on complex formation. The difference in the results from the two techniques is due to the compound effect of the small radius of gyration (*R*_g_) of DNA and its high X-ray scattering length density, almost double that of protein. The SAXS and SANS results at different contrasts indicate that the centres of mass of the DNA and the KorB protein are coincident, with the (‘lighter’) protein outside the (‘denser’) DNA, giving rise to an overall reduction in the observed *R*_g_ value in SAXS (see Supplementary Discussion).

**Figure 5.**
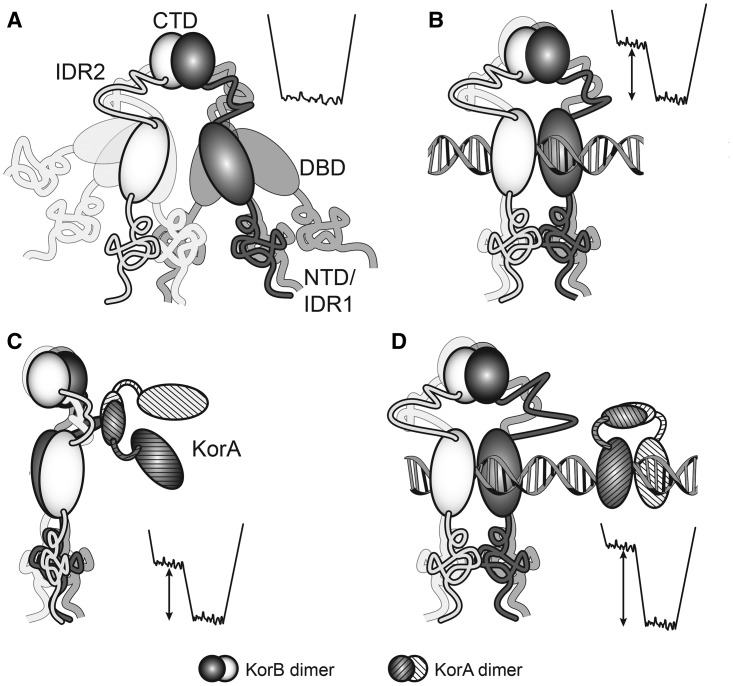
Cartoon of KorB and its complexes with a sketch of the co-operative binding process as applied to intrinsically disordered proteins. Left — cartoon representation of the protein and complexes and right — schematic energy level diagrams. (**A**) Free KorB — the two subunits are white and grey, with ellipsoids for the ordered domains and ribbons for the intrinsically disordered regions, IDR1 and IDR2. The N-terminal domains (NTDs) show some order. DBD, central, DNA-binding domain; CTD, C-terminal dimerisation domain. Multiple conformations are indicated by the fainter shapes. There is one broad energy minimum. (**B**) Protein–DNA complex, KorB as in (**A**) with DNA (rod); the DNA-binding domains of KorB are fixed on the DNA, but the other regions remain mobile. Many conformations are now restricted due to steric hindrance, as shown by a proportion of the conformations having a higher energy, making them unfavourable. The global energy minimum of the protein is narrower compared with (**A**), with an energy barrier. Thus, the remaining conformers are selected by exclusion of the compact conformers from the system, rather than selection of favourable conformers. (**C**) Protein–protein complex of KorB drawn as in (**A**) with KorA (shaded). Here, a similar situation arises to (**B**) whereby the binding of a protein partner sterically restricts the conformers in solution to a similar extended set to that found in (**B**). (**D**) Ternary complex of KorB drawn as in (**A**), DNA (rod) and KorA (shaded). The energy-level diagram is similar to that in (**B**) and (**C**) but with a greater energy barrier. If the selected conformers in situations (**B**) and (**C**) are those that bind the third partner, positive co-operativity is observed.

For the (NΔ150)KorB–O_B_ complex, the effect of DNA binding on the *R_g_* of the protein determined by SAXS is less than that for the full-length protein, even though the protein is smaller. This shows that the centre of mass of the truncated protein is now away from that of the DNA. Using the radii of gyration of the protein and complexes calculated from the SAXS and SANS data and that estimated for the DNA, the centres of masses of the two components are estimated to be ∼30 Å apart (see Supplementary Discussion). This is consistent with the pairwise distance distribution *P*(*r*) calculations of the protein in the (NΔ150)KorB complexes, which suggest that the structured domains are on average ∼78 Å apart, so that the centre of mass of this protein is ∼26 Å from the DNA. As the centre of mass of the full-length KorB is coincident with that of the DNA, the N-terminal domains of the full-length protein must lie on the opposite side of the DNA to the C-terminal domains and the protein must encircle the DNA (Figure 5B). This is consistent with the DNA footprint of the protein covering both strands of the DNA [9]. The difference in *D*_max_ between the full-length protein and (NΔ150)KorB further suggests that the N-terminal 150 amino acids can occupy ∼75 Å. Thus, the SANS measurements, along with the known crystal structures of the DNA-binding and C-terminal domains, allow a general model of the ensemble average distances between the domains in the DNA complex; however, the technique does not allow a more detailed model of the conformations due to their intrinsic disorder.

### The KorB–KorA binary complex and the basis of co-operativity between KorA and DNA binding

We have previously shown by NMR spectroscopy that the C-terminal domain of KorA interacts directly with KorB [14], but the exact region of KorB that is involved is not known. The NMR spectra in this study show that on KorA binding, as with DNA binding, the NMR signals from the DNA-binding domain of KorB broaden before those of the N-terminal domain. This shows that KorA interacts with the DNA-binding domain of KorB. The same region of KorB has been implicated in binding to IncC, by deletion analysis [41].

The CD and SANS data of the KorB/KorA complex and the KorB/DNA complexes show that there is no change in the secondary structure of KorB on complex formation, but that there is an increase in *R*_g_, over that in free KorB (Table 1), demonstrating that an extension of the protein occurs in both complexes. On the formation of the ternary KorB/KorB/O_A_O_B_ complex again, there is little change in the secondary structure of KorB, and it has a similar *R*_g_ value to that in the respective binary complexes. While there is a change in the CD spectrum of the DNA on adding each protein, suggesting bending, the changes observed with KorA and KorB are additive on ternary complex formation, rather than co-operative, raising the question with regard to how the co-operative binding to the DNA occurs. We propose that the positive co-operativity arises by restricting the conformational range of the protein on forming each complex, so that the entropic cost of forming the ternary complex is less than that of forming each binary complex.

We have previously shown that free KorB exists in the complete range of conformers possible for the intrinsically disordered regions (Figure 5A) [21]. On binding DNA, the relative orientation of the central, DNA-binding, domains within the KorB dimer must become fixed on the DNA template on binding. In addition, steric hindrance by the DNA and electrostatic effects provides a barrier to some conformations for the remainder of the protein, and all of these factors reduce the conformational range available to the protein (Figure 5B), preventing collapse of the complex into the more compact conformers seen in the free protein. This leads to the greater prevalence of longer conformers seen in SANS data of the KorB–O_B_ complex. As with DNA binding, KorA binding must give steric and electrostatic effects that reduce the conformational range of KorB, leading to the selection of a subset of longer conformers, as observed in the increased *R*_g_ (Figure 5C). Thus, the binding of either DNA or KorA to KorB changes the conformational ensemble of the protein from a random set in the free protein to a subset of conformers. The subset of conformers in each binary complex is likely to differ, but is more similar to the conformational ensemble in the complex of the other partner than to the wider set of conformers in the free protein. Hence, it is more favourable to form the ternary complex than the binary complexes (Figure 5B,C). This general model of co-operativity does not require protein folding on binding partners, just a reduction in entropy due to steric or electrostatic effects, and intrinsic disorder within the complex may persist, as seen in the ternary KorA/KorB/O_A_O_B_ complex. The degree of co-operativity observed will depend on the extent of conformational restriction in each of the complexes, and the similarity of the restricted conformer set to that favoured by the third partner in its respective binary complex. Hence, as discussed by Hilser and colleagues [43], intrinsic disorder within a protein can be used not only for conformational flexibility for binding single macromolecular partners, such as DNA or proteins, but also as a key mediator of co-operative binding to several partners.

## Abbreviations

CD: circular dichroism
DBD: DNA-binding domain
IDRs: intrinsically disordered regions
SANS: small-angle neutron scattering experiments
SAXS: small-angle X-ray scattering
SEC: size exclusion chromatography
WT: wild type
