# Supplementary material for: Intrinsic disorder in the partitioning protein KorB persists after co-operative complex formation with operator DNA and KorA
Source: Biochem J. 2017 Aug 31;474(18):3121–35. doi: 10.1042/BCJ20170281 (PMC5577506; doi:10.1042/BCJ20170281)
Supplement: Supplementary Table and Figures [file BCJ-474-3121-s1.pdf]

Supplementary information:

Table S1: Analysis of the secondary structure of KorB and KorA from the CD data.

Protein	% α-Helix	% β-Strand	% Turn	% Unordered	Helix segments [a]	Average Helix length	Strand segments [b]	Standard Deviation
**KorB (This study)**	37.3	12.1	21.4	29.3	15.6 (4.1)	9	11.4 (3)	0.015
KorB (Rajasekar et al. 2010)	35	14-17	16-19	28-36				
**KorA (This study)**	70.9	6.2	8.9	14.0	5.2 (5.2)	13.6	0.9 (0.9)	0.039
**KorA (**Rajasekar et al. 2016)[c]	64	9	8	18	5	13	1	

Secondary structure estimate from CD data using the CONTINLL algorithm using SP37 data base set of 37 soluble proteins.
a. In parenthesis- the number of helix segments per 100 amino acid residues (*aa*).
b. In parenthesis -the number of strand segments per 100 *aa*. For both proteins, the length of the strands was 4*aa*
c. Estimated from the PDB entry 5CKT using the DSSP server.

Figure S1: Full ^{15}N-^{1}H TROSY spectra of $^{15}N/^{2}D$ KorB and its complexes taken at 800 MHz.

a Spectrum of $^{15}N/^{2}D$ KorB (~ 0.12 mM dimer) in the presence of 0.75 molar equivalent O_B DNA (red), overlaid on the spectrum of the free KorB. Boxes A and B show four peaks from different regions of the protein while box C shows the tryptophan indole NH peaks.

b $^{15}N/^{2}D$ KorB in the presence of 2 molar equivalents unlabelled KorA (blue), overlaid on the spectrum of the free KorB (black). Boxes A, B and C as above.

c $^{15}N/^{2}D$ KorB in the presence of 1 equivalent unlabelled KorA and 1 equivalent of O_AO_B DNA (green) overlaid with the spectrum of ^{15}N-labelled C-terminal domain (298-358) of KorB (grey) taken at 500 MHz. Box A and B as above

Figure S1

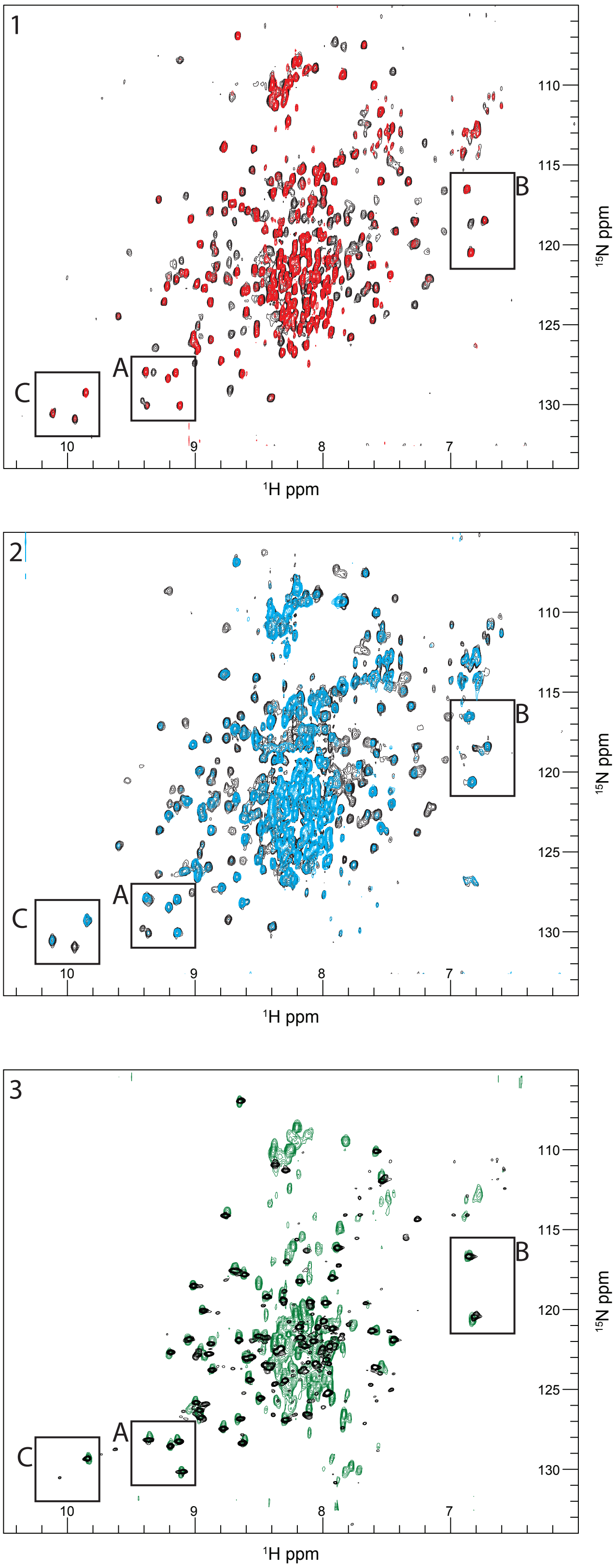

1
A
B
C
110
115
120
125
130
10
9
8
7
^{1}H ppm
^{15}N ppm
2
A
B
C
110
115
120
125
130
10
9
8
7
^{1}H ppm
^{15}N ppm
3
A
B
C
110
115
120
125
130
10
9
8
7
^{1}H ppm
^{15}N ppm

Figure S2: Small angle scattering studies of ^{1}H KorB and ^{2}D KorB.

a. Plots of log *I(q)* vs the scattering vector, *q*, from SAXS studies of KorB in H_2O (purple, circles) and from SANS studies of KorB in 100% $^{2}D_2O$ (blue, diamonds) and H_2O (green, triangles), where *I(q)* is the scattering intensity at *q*. The points show the data, with error bars. The lines show the fits to the scattering calculated from the pairwise distribution functions.

b. Guinier plots of $ln_e\ I\ (q)\ vs\ q^2$ for the data in (a), and also for ^{2}D- KorB in H_2O (grey, triangles) where I(q) is the scattering intensity at *q*. The error bars are smaller than the points. The linearity of the data show that the protein is not aggregated; however the SANS data were not collected at sufficiently low angles to obtain a Guinier plot below qR_g = 1.3.

c) Plot of the square root of the forward scattering intensity ($\sqrt{I_{(0)}}$) *vs* (v/v) % D_2O in the solvent for samples of ^{2}D KorB, giving the match point ~ 100% D_2O. The linearity of the plot shows that the conformation of the protein is not affected by deuteration of the solvent.

d). Plots of the pairwise distribution functions *(P(r) vs r)* of ^{1}H KorB from SAXS (purple, circles), or from SANS in H_2O (green triangles) or D_2O (blue, diamonds), and of ^{2}D KorB (grey, triangles) from SANS in H_2O.

Figure S2

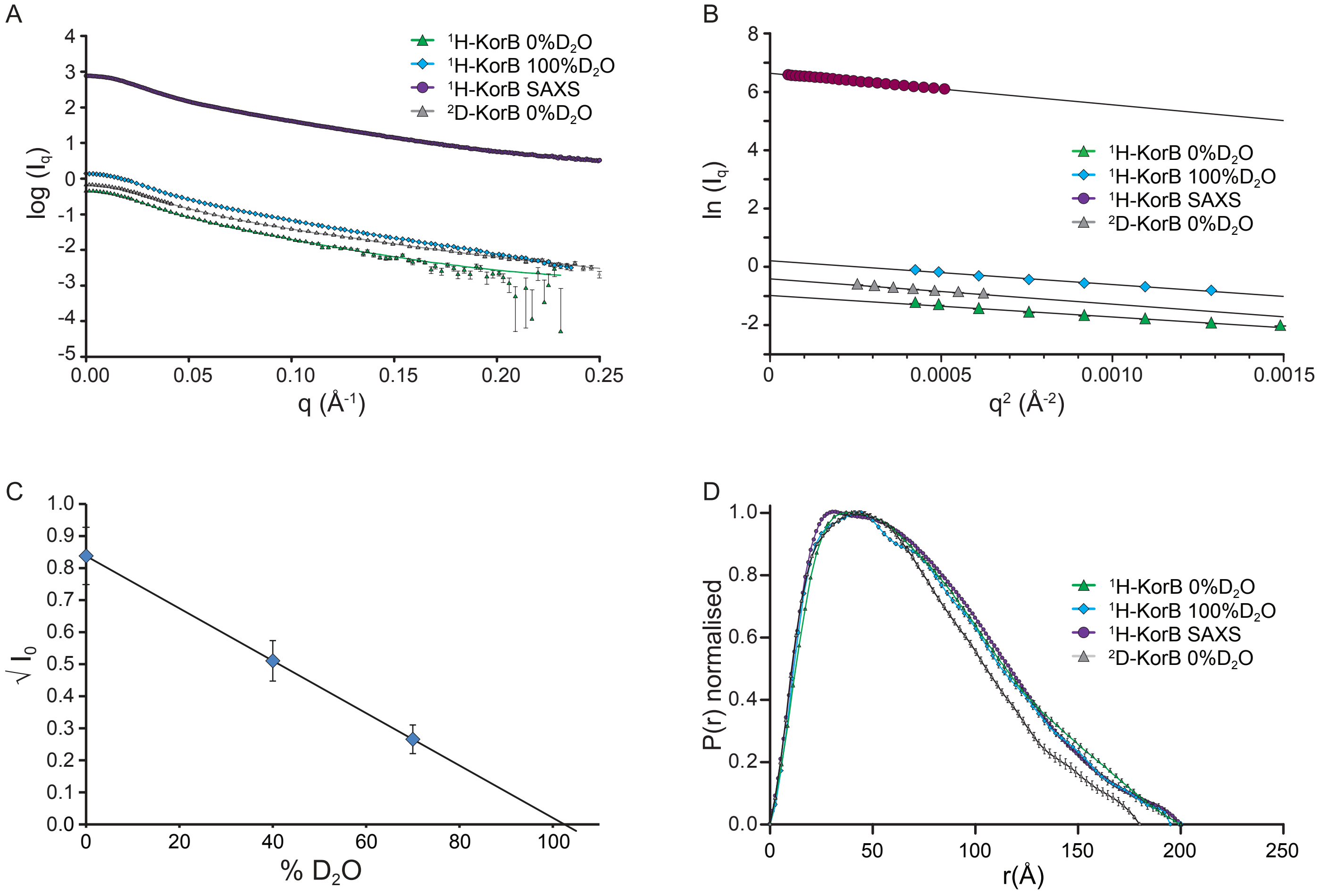

A
B
C
D
log (Iq)
q (Å-1)
ln (Iq)
q2 (Å-2)
√I0
% D2O
P(r) normalised
r(Å)
1H-KorB 0%D2O
1H-KorB 100%D2O
1H-KorB SAXS
2D-KorB 0%D2O

Figure S3- SANS contrast data for KorB-O_B and KorB-KorA complexes –

a. plot of the square root of the forward scattering intensity ($\sqrt{I(0)}$) *vs* (v/v) % D_2O in the solvent for KorB-O_B. The linearity of the plot show that the complexes are not affected by deuteration of the solvent

b. Guinier plot of $ln_e\ I(q)\ vs\ q^2$ for KorB-O_B in H_2O (green, triangles), in D_2O (blue, diamonds) and in 65% D_2O (red, squares). The error bars are smaller than the points. The linearity of the plot show that the complexes are single species and not aggregated.

Figure S3

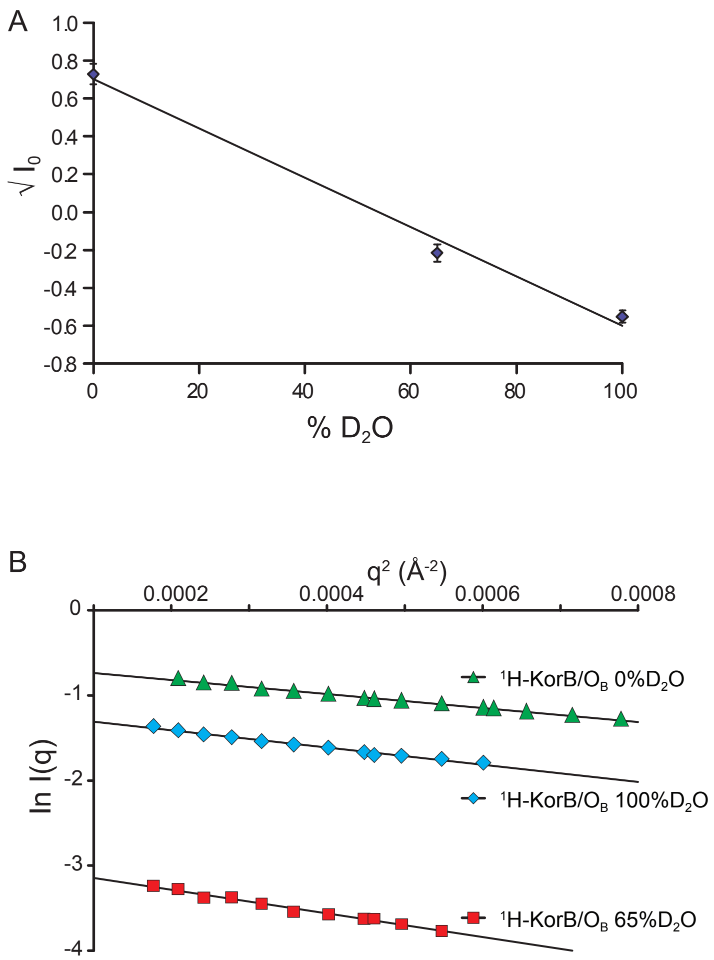

A
1.0
0.8
0.6
0.4
0.2
0.0
-0.2
-0.4
-0.6
-0.8
√ I0
0
20
40
60
80
100
% D2O
B
q2 (Å-2)
0.0002
0.0004
0.0006
0.0008
0
-1
-2
-3
-4
ln I(q)
1H-KorB/OB 0%D2O
1H-KorB/OB 100%D2O
1H-KorB/OB 65%D2O

Figure S4- SANS contrast data for KorB-O_AO_B complexes –

a. plot of the square root of the forward scattering intensity ($\sqrt{I_{(0)}}$) *vs* (v/v) % D_2O in the solvent for samples of 46% ^2D KorA, giving the solvent match point at ~ 64% D_2O.

b. plot of the square root of the forward scattering intensity ($\sqrt{I_{(0)}}$) *vs* (v/v) % D_2O in the solvent for samples of KorB- 46%^2D-KorA O_AO_B complexes. The linearity of the plot shows that the complex is not affected by deuteration of the solvent.

Figure S4

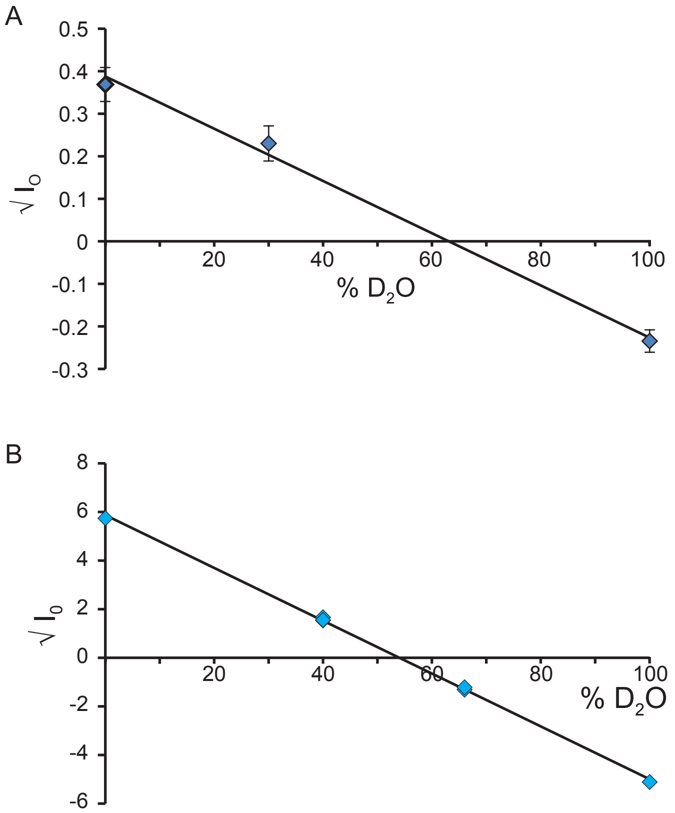

A
0.5
0.4
0.3
0.2
0.1
0
-0.1
-0.2
-0.3
20
40
60
80
100
% D2O
√ IO
B
8
6
4
2
0
-2
-4
-6
20
40
60
80
100
% D2O
√ I0

Supplementary discussion-

Estimation of distances within the protein-DNA complex.

For an inhomogeneous scattering particle, such as a DNA-protein complex, where each component has a different scattering length density, the radius of gyration observed is given by the formula:

$$R_g^2 = R_{g,1}^2 x_1 + R_{g,2}^2 (1-x_1) + L_{12}^2 x_1 (1-x_1) \quad (1)$$

where the index 1 is for protein and 2 is for DNA. L_{12} is the distance between the centres of mass of the two components and x_1 is the fraction of scattering intensity from component 1 in the complex. As the values of R_g and L_{12} are squared, the calculations are sensitive to small errors, in addition the R_g observed depends on the hydration layer. Since the scattering intensity of the protein at 65% D_2O is very low, we have used the SANS data in H_2O and D_2O, together with the scattering lengths of the protein in these solvents, estimated by the program MULCh (34) to give approximate values of L_{12} as below.

The radius of gyration of the DNA can be considered to be that of a rod of length 88.4 Å, radius 10 Å, i.e. $R_{g,2}$ = 26.5 Å. Using this value, and the R_gs of the KorB-O_B complex measured in SANS experiments in H_2O and D_2O, where x_1 ~ 0.80, and 0.89, respectively; we find that L_{12} is zero i.e. the centre of mass of the protein is coincident with that for the DNA. The R_g for the protein in the complex from these two SANS measurements is 56.1 Å and 59.9 Å respectively; a little lower than the 62.6 +/- 1.7 Å estimated from the *P(r)* distribution in 65% D_2O. In SAXS, x_1 ~ 0.78; using the average calculated R_g for the protein and the DNA, the expected R_g for the complex is 52.7 Å, slightly greater than the measured value, 50.7 Å, confirming the low value for L_{12}.

Doing a similar calculation as above for the (NΔ150)KorB-O_B complex, using the SANS results in H_2O and D_2O, where x_1 ~ 0.72 and 0.84 respectively, the R_g of the (NΔ150)KorB within the complex is estimated to be 40.3 Å and 40.1 Å respectively, again a little lower than the estimate from the the *P(r)* distribution in 65% D_2O, and L_{12} is again very small. If we use this R_g value for (NΔ150)KorB with the

SAXS data, where x_1 = 0.65; the centre of mass of the DNA and that of the truncated KorB protein, L_{12}, are now estimated to be approximately 30 Å apart.

(NΔ150)KorB is made of two domains- the central domain of 100 residues and the C-terminal domain of 60 residues, with a highly flexible linker of 40 amino acids. If the centre of mass of the DNA-binding domain is coincident with that of the DNA, while the flexible linker is evenly distributed around the centre of mass of the complex, the centre of mass of the C-terminal domain will be 50 Å from the centre of mass of the complex; so the two domains will be ~ 80 Å apart
